# Legal changes and evidence on unmet need for contraception, Philippines

**DOI:** 10.2471/BLT.23.290577

**Published:** 2024-10-02

**Authors:** Miguel Antonio Garcia Estrada, Kent Jason Go Cheng, Rutcher Madera Lacaza

**Affiliations:** a Department of Public Administration and Policy, University of Georgia, Athens, United States of America (USA).; b Center for Healthy Aging, Pennsylvania State University, University Park, USA.; c School of Statistics, University of the Philippines, T M Kalaw Street, Diliman, Quezon City, Metro Manila, 1101, Philippines.

## Abstract

**Objective:**

To investigate the relationship between the Responsible Parenthood and Reproductive Health Law in the Philippines and women’s unmet needs for contraception.

**Methods:**

The study involved data on women aged 18 to 49 years from the 2013 (*n* = 14 053), 2017 (*n* = 21 835) and 2022 (*n* = 24 253) Philippine Demographic and Health Surveys. The Responsible Parenthood and Reproductive Health Law was enacted in 2012, but not fully implemented until 2017. Survey-weighted logistic regression was used to estimate the association between variables and an unmet need for contraception, and the probability that women in different wealth quintiles would have an unmet need.

**Findings:**

We observed a persistent gap in unmet needs between women in the lowest and highest wealth quintiles in all years. In 2013, the odds of unmet needs for women in the lowest quintile compared with those in the highest were 1.288 (standard error (SE): 0.124); and in 2022, it was 1.287 (SE: 0.113). Nevertheless, the weighted proportion of women with unmet needs declined between 2013 and 2022; in the lowest wealth quintile, it fell from 18.4% to 10.6%. Moreover, the probability of having an unmet need declined across all wealth quintiles between 2013 and 2022; the largest decline was from 0.146 (95% confidence interval, CI: 0.131–0.162) to 0.088 (95% CI: 0.079–0.098) in the lowest quintile.

**Conclusion:**

The unmet needs for contraception declined substantially following implementation of a new reproductive health law. However, there was a persistent gap in unmet needs between the lowest and highest wealth quintiles.

## Résumé

**Objectif:**

Étudier la relation entre la loi sur la parentalité responsable et la santé reproductive des Philippines d’une part et les besoins non satisfaits des femmes en matière de contraception d’autre part.

**Méthodes:**

L’étude a porté sur des données relatives à des femmes âgées de 18 à 49 ans provenant des enquêtes démographiques et sanitaires philippines de 2013 (*n* = 14 053), 2017 (*n* = 21 835) et 2022 (*n* = 24 253). La loi sur la parentalité responsable et la santé reproductive a été promulguée en 2012, mais n’a été pleinement mise en œuvre qu’en 2017. Une régression logistique pondérée par l’enquête a été appliquée pour évaluer l’association entre les variables et un besoin non satisfait en matière de contraception et la probabilité que les femmes appartenant à différents quintiles de richesse soient confrontées à un besoin non satisfait.

**Résultats:**

Nous avons observé un écart persistant dans les besoins non satisfaits entre les femmes appartenant aux quintiles de richesse les plus bas et celles appartenant aux quintiles les plus élevés, quelle que soit l’année. En 2013, la probabilité de besoins non satisfaits pour les femmes du quintile le plus bas par rapport à celles du quintile le plus élevé était de 1,288 (écart type: 0,124), et en 2022, elle était de 1,287 (écart type: 0,113). Néanmoins, la proportion pondérée de femmes ayant des besoins non satisfaits a diminué entre 2013 et 2022: dans le quintile de richesse le plus bas, elle est passée de 18,4% à 10,6%. En outre, la probabilité d’avoir un besoin non satisfait a diminué dans tous les quintiles de richesse entre 2013 et 2022: la baisse la plus importante était de 0,146 (intervalle de confiance à 95% (IC): 0,131 à 0,162) à 0,088 (IC à 95%: 0,079-0,098) dans le quintile le plus bas.

**Conclusion:**

Les besoins non satisfaits en matière de contraception ont considérablement diminué à la suite de la mise en œuvre d’une nouvelle loi sur la santé reproductive. Toutefois, un écart persiste entre les quintiles de richesse les plus bas et les plus élevés.

## Resumen

**Objetivo:**

Investigar la relación entre la Ley de Paternidad Responsable y Salud Reproductiva de Filipinas y las necesidades insatisfechas de anticoncepción de las mujeres.

**Métodos:**

El estudio incluyó datos de mujeres de 18 a 49 años de las Encuestas demográficas y de salud de Filipinas de 2013 (*n* = 14 053), 2017 (*n* = 21 835) y 2022 (*n* = 24 253). La Ley de Paternidad Responsable y Salud Reproductiva se promulgó en 2012, pero no se aplicó en su totalidad hasta 2017. Se utilizó una regresión logística ponderada por la encuesta para estimar la asociación entre las variables y una necesidad insatisfecha de anticoncepción y la probabilidad de que las mujeres de diferentes quintiles de riqueza tuvieran una necesidad insatisfecha.

**Resultados:**

Se observó una diferencia persistente en las necesidades insatisfechas entre las mujeres de los quintiles de riqueza más bajos y más altos en todos los años. En 2013, las probabilidades de necesidades insatisfechas para las mujeres del quintil más bajo en comparación con las del más alto eran de 1288 (error estándar, EE: 0,124) y, en 2022, de 1287 (EE: 0,113). No obstante, la proporción ponderada de mujeres con necesidades insatisfechas disminuyó entre 2013 y 2022; en el quintil de riqueza más bajo, se redujo del 18,4% al 10,6%. Además, la probabilidad de tener una necesidad insatisfecha disminuyó en todos los quintiles de riqueza entre 2013 y 2022; la mayor disminución fue de 0,146 (intervalo de confianza del 95%, IC: 0,131-0,162) a 0,088 (IC del 95%: 0,079-0,098) en el quintil más bajo.

**Conclusión:**

Las necesidades insatisfechas de anticonceptivos disminuyeron notablemente tras la aplicación de una nueva ley en materia de salud reproductiva. Sin embargo, persiste una diferencia de necesidades insatisfechas entre los quintiles de riqueza más bajos y más altos.

## ملخص

الغرض التحقيق في العلاقة بين "قانون الأبوة المسؤولة والصحة الإنجابية" في الفلبين من جهة، واحتياجات السيدات غير المستوفاة من وسائل منع الحمل.

**الطريقة:**

شملت الدراسة بيانات عن السيدات في سن 18 إلى 49 عامًا من المسوح السكانية والصحية الفلبينية لعام 2013 (ن = 14053)، و2017 (ن = 21835)، و2022 (ن = 24253). صدر "قانون الأبوة المسؤولة والصحة الإنجابية" في عام 2012، ولكن لم يتم تنفيذه بالكامل حتى عام 2017. تم استخدام التحوّف اللوجستي القائم على المسح، لتقدير الارتباط بين المتغيرات والاحتياج غير المستوفى من وسائل منع الحمل، واحتمال أن يكون هناك احتياج غير مستوفى لدى السيدات في الفئات الخمسية السكانية المختلفة للثروة.

**النتائج:**

لاحظنا وجود فجوة دائمة في الاحتياجات غير المستوفاة بين السيدات في أدنى وأعلى فئات خمسية سكانية للثروة في جميع الأعوام. في عام 2013، كانت احتمالات الاحتياجات غير المستوفاة للسيدات في أدنى فئة خمسية سكانية مقارنة بتلك الموجودة في أعلى فئة خمسية سكانية 1.288 (SE: 0.124)، وفي عام 2022، كانت 1.287 (SE: 0.113). وعلى الرغم مع ذلك، انخفضت النسبة المرجحة للسيدات من أصحاب الاحتياجات غير المستوفاة بين عامي 2013 و2022؛ ففي أدنى فئة خمسية سكانية للثروة، انخفضت من %18.4 إلى %10.6. وبالإضافة إلى ذلك، انخفضت احتمالية وجود احتياج غير مستوفى عبر جميع الفئات الخمسية السكانية للثروة بين عامي 2013 و2022؛ كان أكبر انخفاض من 0.146 (بفاصل ثقة مقداره %95: 0.131 إلى 0.162) إلى 0.088 (بفاصل ثقة مقداره %95: 0.079 إلى 0.098) في أدنى فئة خمسية سكانية.

**الاستنتاج:**

انخفضت الاحتياجات غير المستوفاة من وسائل منع الحمل بشكل ملموس بعد تطبيق قانون الصحة الإنجابية الجديد. ومع ذلك، كانت هناك فجوة دائمة في الاحتياجات غير المستوفاة بين أعلى وأدنى فئات خمسية سكانية للثروة.

## 摘要

**目的:**

旨在探讨菲律宾《负责任父母与生殖健康法》与女性的未满足避孕需求之间的关系**。**

**方法:**

本研究采用了通过 2013 年 (*n* = 14,053)、2017 年 (*n* = 21,835) 和 2022 年 (*n* = 24,253)《菲律宾人口与健康调查》所收集的 18 至 49 岁女性的数据。《负责任父母与生殖健康法》颁布于 2012 年，但直至 2017 年才得以全面实施。我们使用了调查加权逻辑回归以估算变量与未满足的避孕需求之间的关联性，以及不同财富五分位数范围内的女性存在未满足需求的概率。

**结果:**

我们发现，在所有年份中，最低和最高财富五分位数范围内的女性在未满足需求方面始终存在差距。2013 年，与最高财富五分位数范围内的女性相比，最低财富五分位数范围内的女性中存在未满足需求的人数占比为 1.288（SE：0.124），而2022 年该比例为 1.287（SE：0.113）。尽管如此，在 2013 年至 2022 年期间，需求未得到满足的女性的加权比例有所下降；这一比例在最低财富五分位数范围内的女性中从 18.4% 降至了 10.6%。此外，在 2013 年至 2022 年期间，对于所有财富五分位数范围内的女性，存在未满足需求的概率都有所下降；降幅最大的是最低财富五分位数范围内的女性，从 0.146（95% 置信区间，CI：0.131–0.162）降至了 0.088（95% CI：0.079–0.098）。

**结论:**

新的生殖健康法开始实施后，未满足的避孕需求大幅下降。但是，最低和最高财富五分位数范围内的女性在未满足需求方面始终存在差距。

## Резюме

**Цель:**

Изучить взаимосвязь между Законом о сознательном родительстве и репродуктивном здоровье на Филиппинах и неудовлетворенными потребностями женщин в контрацепции.

**Методы:**

В исследовании использовались данные о женщинах в возрасте от 18 до 49 лет, полученные в ходе проведения демографических и медицинских обследований на Филиппинах в 2013 (n = 14 053), 2017 (n = 21 835) и 2022 (n = 24 253) годах. Закон о сознательном родительстве и репродуктивном здоровье был принят в 2012 году, но полностью вступил в силу только в 2017 году. Для оценки связи между переменными анализа и неудовлетворенной потребностью в контрацепции, а также вероятности того, что женщины из разных квинтилей благосостояния будут иметь неудовлетворенную потребность, использовалась взвешенная по результатам опроса логистическая регрессия.

**Результаты:**

На протяжении всех лет наблюдался постоянный разрыв в уровне неудовлетворенных потребностей между женщинами из квинтилей с самым низким и самым высоким уровнем благосостояния. В 2013 году коэффициент неудовлетворенных потребностей для женщин из низшего квинтиля по сравнению с женщинами из высшего квинтиля составлял 1,288 (SE: 0,124), а в 2022 году – 1,287 (SE: 0,113). Тем не менее взвешенная доля женщин с неудовлетворенными потребностями снизилась в период с 2013 по 2022 год. В квинтиле с самым низким уровнем благосостояния она сократилась с 18,4 до 10,6%. Более того, вероятность наличия неудовлетворенных потребностей снизилась во всех квинтилях благосостояния в период с 2013 по 2022 год; наибольшее снижение произошло с 0,146 (95%-й ДИ: 0,131–0,162) до 0,088 (95%-й ДИ: 0,079–0,098) в самом низком квинтиле.

**Вывод:**

Неудовлетворенные потребности в контрацепции значительно снизились после введения нового закона о репродуктивном здоровье. Однако сохранялся разрыв в неудовлетворенных потребностях между квинтилями с самым низким и самым высоким уровнем благосостояния.

## Introduction

Access to reproductive health services is integral to an individual’s well-being and is recognized as a key element of the 2030 Agenda for Sustainable Development.^1^ One target under sustainable development goal 3 is to ensure universal access to sexual and reproductive health services, with an emphasis on women’s needs for family planning. This target has figured prominently in both policy and academic discussions on promoting an individual’s right to meet their reproductive health goals and, more broadly, on achieving socioeconomic development.^2^^,^^3^

The proportion of women with an unmet need for contraception is a key indicator of access to reproductive health services. The indicator provides an assessment of the extent to which women who prefer not to be pregnant are unable to use contraceptives.^4^ For many years, Demographic and Health Surveys (DHS) have regularly estimated the proportion of women of reproductive age with unmet needs for contraception. Although the measure has been criticized, it has been useful for health policy and programming.^5^ Both reducing women’s unmet needs for contraception and increasing contraceptive use are associated with lower maternal mortality and improvements in women’s health.^6^^,^^7^ Hence, an understanding of policies that guarantee universal access to reproductive health services is important for tackling the persistently high proportion of women with unmet needs for contraception, particularly in developing countries.^8^

The Philippines is a lower-middle-income country in the World Health Organization (WHO) Western Pacific Region that had a population of 110 million in 2020, about 40% of whom were women of reproductive age (i.e. between 15 and 49 years of age).^9^ Family planning in the country is juxtaposed between modern and traditional methods and, for a long time, the adoption of a national policy guaranteeing access to modern contraceptives has been opposed by certain influential groups.^10^^,^^11^ However, after three decades of debate, in December 2012 the Congress of the Philippines passed the Responsible Parenthood and Reproductive Health Law (Republic Act no. 10354), which codified universal access to reproductive health services, including non-abortifacient contraceptives.^12^ This law provides for family planning services that include: (i) increasing women of reproductive age’s understanding of their menstrual cycles; (ii) providing information on maternal, infant and child health and nutrition; (iii) providing adolescent and youth reproductive health counselling; and (iv) preventing and treating sexually transmittable infections.

Enactment of the Responsible Parenthood and Reproductive Health Law was ground-breaking in a country where government-supported access to modern contraceptives remains controversial. Consequently, the policy’s early years were met with legal and administrative challenges. At the outset, the law’s planned implementation in January 2013 was halted by a constitutional challenge at the Supreme Court. Although the Court upheld the law in 2014, except for eight provisions, in 2015, it issued a restraining order on certain aspects of the law following a petition against reproductive health policy.^13^ The law was not fully implemented until January 2017, when the President issued Executive Order No. 12, 2017,^14^ which aimed to achieve zero unmet needs for family planning. Hence, between 2014 and 2017, legal challenges prevented the implementation of some aspects of the law, particularly those concerning contraceptive implants and the registration of previously approved contraceptives.

**Fig. 1:**
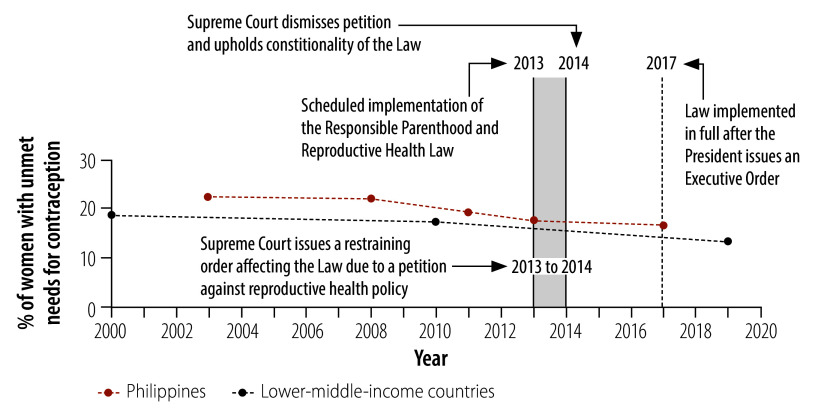
Timeline for implementation of the Philippines’ Responsible Parenthood and Reproductive Health Law and changes in unmet needs for contraception in the Philippines and lower-middle-income countries, 2000–2020

Notes: The graphs were based on 2024 data on unmet needs for contraception from the World Bank.^15^ Women were aged between 18 and 49 years.

Fig. 1 presents a timeline for the adoption and implementation of the law, accompanied by graphs illustrating the overall trend in unmet needs for contraception in the country and in lower-middle-income countries on average. Despite a general decline in women’s unmet needs for contraception in the Philippines from 2003 to 2017, the level remained higher than in lower-middle-income countries overall. This difference highlights the necessity of bolder efforts to address the high rate of unmet needs for contraception.

Although 99% of Filipino women had some knowledge of modern contraceptive methods around the time when the law was enacted, a substantial proportion continued to have an unmet need for contraception.^16^ In the past decade, there has been a considerable decline in women’s unmet needs for contraception globally.^15^ Between 2010 and 2020, the decline was pronounced in lower-middle-income countries: there was an average drop of 5.5 percentage points (from 19.0% to 13.5%) in lower-middle-income countries compared with 2.6 percentage points (from 19.3% to 16.7%) in the Philippines.^15^ Moreover, the unmet need for contraception in the Philippines varied according to socioeconomic status: women without a formal education were almost twice as likely to have an unmet need compared with those who finished college.^16^ A similar disparity was observed between women in the lowest and highest wealth quintiles.

The aim of our study was to examine the relationship between the introduction of the Philippines’ landmark reproductive health policy and women’s access to contraceptives, with particular reference to how women’s unmet needs for contraception changed during the first decade following the enactment of the Responsible Parenthood and Reproductive Health Law in 2012. We explored one key question: Did unmet needs for contraception decline across all socioeconomic groups between 2013 and 2022? In addition, we investigated differences in the pattern of unmet needs in 2013, 2017 and 2022 – three years that were critical points in the law’s implementation.

## Methods

Our analysis involved nationally representative survey data on women aged 15 to 49 years from the 2013, 2017 and 2022 DHSs for the Philippines, which contain the most recent publicly available data for the country.^16^^–^^18^ The surveys were conducted by the Philippines Statistics Authority with technical guidance from the United States Agency for International Development (USAID).

The main outcome of interest was a woman’s unmet need for contraception – a binary variable with a value of 1 if a woman reported having any unmet need and a value of 0 otherwise. We adopted USAID’s 2012 revised definition of an unmet need for contraception,^4^ which simplified several qualifying conditions and applied only to women with partners. Generally, a woman had an unmet need for contraception if she did not wish to have a child or more children but was not using any contraceptive method.

The main independent variable was wealth, which was divided into quintiles based on a wealth index determined in each DHS.^19^ The lowest quintile was coded as 1 and the highest was coded as 5. The analytical model controlled for: (i) age (18–24 years; 25–34 years; and 35–49 years); (ii) place of residence (1 for urban and 0 for rural); (iii) number of children younger than 5 years; and (iv) region of residence (a fixed-effects variable was set according to which of the 17 administrative regions in the Philippines the woman lived in). Age has implications for a woman’s fecundity and for her contraceptive choices because older women have a lower rate of contraception failure.^20^^,^^21^ Recent studies in developing countries highlight how certain social groups, such as young women and women with a lower socioeconomic status, can be at a disadvantage in accessing contraceptives.^22^^–^^24^ Moreover, a positive association has been reported between the number of young children a woman has and an unmet need for contraception.^25^ Conversely, residing in an urban area can mean closer proximity to family planning services and may be negatively associated with an unmet need for contraception.^26^

We constructed an index for media exposure using a principal component analysis: respondents were divided into quintiles based on their exposure to newspapers, radio and television, and to family planning information from any of these sources. The index value increased with the number of different media a woman was exposed to weekly. Exposure to information about family planning has been shown to be important in situations where contraception uptake is low.^3^^,^^27^

Survey-weighted logistic regression was used to estimate the odds that a particular variable was associated with an unmet need for contraception and the probability that a woman in a particular wealth quintile would have an unmet need. All analyses were performed using Stata 18.0 (StataCorp LLC, College Station, United States of America).

## Findings

Our original sample consisted of 16 155 respondents from the 2013 survey, 25 074 from the 2017 survey and 27 821 from the 2022 survey. However, in line with the Responsible Parenthood and Reproductive Health Law, which requires minors younger than 18 years to obtain written consent from a parent or guardian if they wish to access modern family planning methods, our sample included only women aged 18 to 49 years: 14 053 from the 2013 survey, 21 835 from the 2017 survey and 24 253 from the 2022 survey. The average age of the women in the reduced sample was 32 years. Around 70% of women in the 2013 and 2017 samples had sexual partners, compared with only 64% in 2022. The mean proportion of women residing in an urban area increased from 53% in 2013 to 63% in 2017, before decreasing to 59% in 2022 (available in authors’ online repository).^28^

Overall, there was no significant difference in women’s unmet needs for contraception across the wealth quintiles during the two early survey years – 2013 and 2017. Among women in the four lower quintiles, only those in the lowest wealth quintile had a significantly higher odds of an unmet need compared with those in the highest quintile in all three survey years (Table 1). In 2013, the odds that a woman in the lowest quintile would have an unmet need compared with a woman in the highest quintile was 1.288 (standard error, SE: 0.124; *P* < 0.01). In 2017, the corresponding odds ratio was 1.151 (SE: 0.089; *P* < 0.1) and, in 2022, it was 1.287 (SE: 0.113; *P* < 0.01). In 2022, about 5 years after the executive order that led to full implementation of the Responsible Parenthood and Reproductive Health Law, the odds that a woman in any of the four lower wealth groups would have an unmet need for contraception compared with a woman in the highest quintile became significantly greater than 1 (Table 1). For example, the odds that a woman in the second quintile would have an unmet need compared with a woman in the highest quintile were 1.302 (SE: 0.104; *P* < 0.01); and the odds for a woman in the fourth quintile compared with a woman in the highest were 1.194 (SE: 0.090; *P* < 0.05). Moreover, although not statistically significant in 2013 and 2017, the odds that a woman in the second, third or fourth quintile would have an unmet need compared with a woman in the highest quintile were generally greater than 1. 

**Table 1:** Variables associated with an unmet need for contraception among women of reproductive age, by logistic regression analysis, Philippines, 2013, 2017 and 2022

Variable^a^	Women’s likelihood of having an unmet need for contraception,^b^ OR (SE)		
	2013 (*n* = 14 020)	2017 (*n* = 21 835)	2022 (*n* = 24 253)
**Wealth, by quintile**			
First	1.288 (0.124)^c^	1.151 (0.089)^d^	1.287 (0.113)^c^
Second	1.023 (0.092)	1.049 (0.076)	1.302 (0.104)^c^
Third	0.963 (0.082)	0.994 (0.068)	1.288 (0.098)^c^
Fourth	1.067 (0.087)	1.001 (0.066)	1.194 (0.090)^e^
Fifth	Reference	Reference	Reference
**Place of residence**			
Rural	Reference	Reference	Reference
Urban	0.961 (0.062)	0.953 (0.048)	0.876 (0.048)^e^
**Media exposure index,^f^ by quintile**			
First	Reference	Reference	Reference
Second	1.162 (0.094)^d^	1.055 (0.066)	0.852 (0.066)^e^
Third	1.067 (0.085)	0.967 (0.070)	0.856 (0.058)^e^
Fourth	1.011 (0.079)	0.924 (0.066)	0.883 (0.062)^d^
Fifth	1.228 (0.116)^d^	1.098 (0.072)	0.891 (0.068)
**Age group, years**			
18–24	Reference	Reference	Reference
25–34	1.206 (0.086)^c^	1.414 (0.087)^g^	1.682 (0.110)^g^
35–49	1.641 (0.111)^g^	2.259 (0.131)^g^	1.652 (0.106)^g^
**No. children aged < 5 years^h,j^**	1.531 (0.040)^i^	1.511 (0.035)^i^	1.686 (0.043)^i^
**Constant^k^**	0.047 (0.005)^i^	0.048 (0.007)^i^	0.045 (0.006)^i^

OR: odds ratio; SE: standard error.

^a^ The analysis included a fixed-effects variable that was set according to which of the 17 administrative regions of the Philippines the woman lived in.

^b^ Women were aged between 18 and 49 years.

^c^ *P* < 0.01 relative to the reference category.

^d^ *P* < 0.1 relative to the reference category.

^e^ *P* < 0.05 relative to the reference category.

^f^ The media index was based on the number of different media a woman was exposed to weekly and the family planning information available through these media.

^g^ *P* < 0.001 relative to the reference category.

^h^ The number of children aged ≤ 5 years was treated as a continuous variable.

^i^ *P* < 0.001.

^j^ The ORs are for each additional child.

^k^ The constant ensured that the mean of the residuals equalled zero. Its ORs do not have any substantive significance for the association with an unmet need for contraception.

**Fig. 2:**
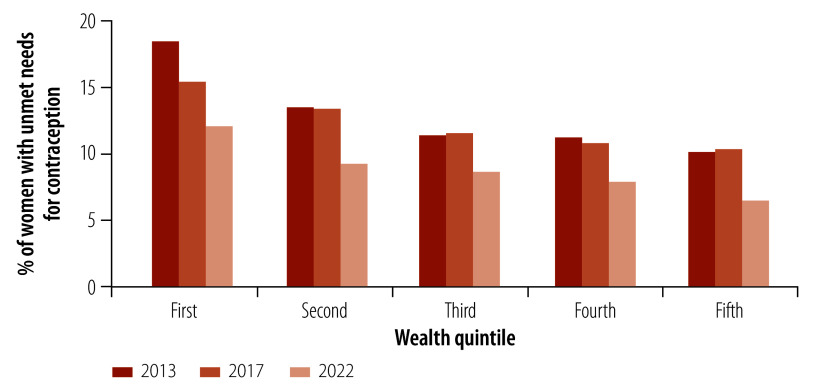
Women with unmet needs for contraception, by wealth quintile, Philippines, 2013, 2017 and 2022

Notes: Women were aged between 18 and 49 years. Data were obtained from the 2013, 2017 and 2022 Demographic and Health Surveys for the Philippines.^16^^–^^18^

A more complete evaluation of the findings entailed looking at time trends in the proportion of women with unmet needs for contraception in each wealth quintile. The proportion of women with unmet needs fell between 2013 and 2022 in all quintiles (Fig. 2). In 2013, an average of 18.6% (514 out of 2761) of women aged 18 to 49 years in the lowest wealth quintile reported having an unmet need compared with 10.1% (310 out of 3065) of corresponding women in the highest quintile. By 2017, the proportion in the lowest quintile had decreased to 15.1% (774 out of 5127), whereas that in the highest stayed roughly the same, at 10.5% (391 out of 3741). By 2022, the proportions declined further to 12.4% (714 out of 5768) in the lowest quintile, and 6.6% (289 out of 4399) in the highest.

**Fig. 3:**
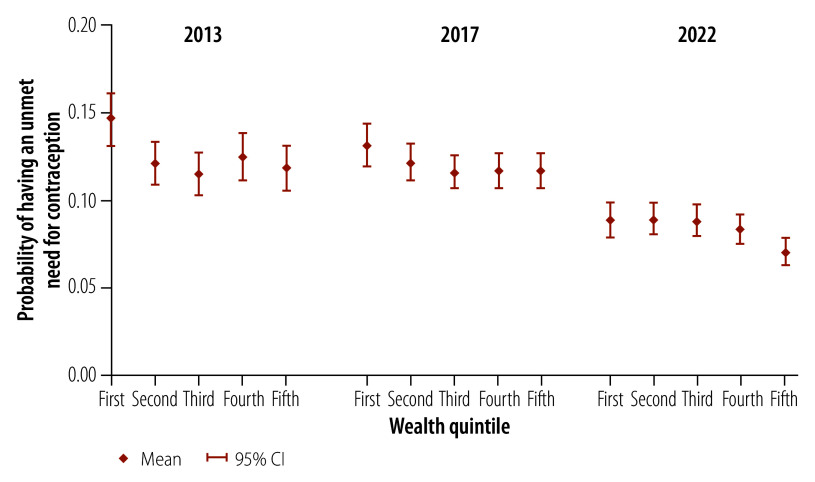
Probability of having an unmet need for contraception among women, by logistic regression analysis, Philippines, 2013, 2017 and 2022

We also calculated the probability that a woman in a specific wealth quintile would have an unmet need for contraception. All estimates of the probability of having an unmet need across all wealth quintiles for the three survey years were significant at the 1% level. We found that the probability of having an unmet need for contraception declined over time for women in all five wealth quintiles (Fig. 3). For a woman in the lowest wealth quintile, the mean probability of having an unmet need dropped from 0.146 (95% confidence interval, CI: 0.131–0.162) in 2013 to 0.088 (95% CI: 0.079–0.098) in 2022 (online repository).^28^ For a woman in the highest quintile, the mean probability declined from 0.118 in 2013 to 0.070 in 2022. In 2013, only women in the lowest quintile had a notably higher probability of an unmet need for contraception than women in the other four quintiles (Fig. 3); women in the second, third, fourth and fifth quintiles all had a mean probability around 0.12. The pattern of probabilities showed minimal changes between 2013 and 2017. However, between 2017 and 2022, there was a clear decrease in the probability of having an unmet need for contraception across all wealth quintiles. In particular, the difference in mean probability between the lowest and highest wealth quintiles decreased significantly from 0.028 (0.146 in the lowest quintile versus 0.118 in the highest) in 2013 to 0.018 (0.088 versus 0.070, respectively) in 2022 (*P* < 0.05).

CI: confidence interval.

Notes: Women were aged between 18 and 49 years.

To address the issue of overlapping 95% CIs for the predicted probability of having an unmet need for contraception across wealth groups, we performed a pairwise comparison of predictive margins by wealth quintile. The results showed that the difference in predicted probability between the lowest and highest wealth quintiles was significant at the 5% level in both 2013 and 2022, with a smaller difference in 2022 (online repository).^28^

The robustness of the logistic regression analysis findings was assessed using different model specifications. The findings were found to be generally stable (online repository)^28^ and not sensitive to the inclusion or exclusion of additional controls, such as the regional fixed-effects variable.

## Discussion

Our study findings indicate that there is a gap in unmet needs for contraception between Filipino women in the lowest and highest wealth quintiles that has persisted for many years. Notably, while the difference between the wealth quintiles, as indicated by odds ratios, appeared unchanged between 2013 and 2022, the absolute proportion of women with unmet needs declined substantially. Hence, our findings actually demonstrate a persistent gap within a declining trend.

There are several possible explanations for the declining trend in unmet needs for contraception and the persistent gap between wealth quintiles. The decline in unmet needs followed implementation of the Responsible Parenthood and Reproductive Health Law, which required close coordination between national government – with the health department as the lead agency responsible for contraceptive procurement and for the implementation of related programmes – and local governments at the forefront of delivering reproductive health services. The Philippines’ Commission on Population and Development facilitated coordination among various government agencies, in line with its mandate. Moreover, full implementation of the law funnelled additional funds into reproductive health services. In 2017, the total funds allocated to the health department’s Family and Responsible Parenthood programme was 4.3 billion Philippine pesos (₱; 86 million United States dollars, US$), which was almost double the ₱2.3 billion (US$ 46 million) allocated in 2016.^29^ This increase enabled the procurement of substantial quantities of modern family planning supplies.

In principle, the persistent gap in unmet needs for contraception between wealth quintiles should have been eliminated by the universal provision of access to family planning services required by the Responsible Parenthood and Reproductive Health Law. Although the observed trends show a clear decline in the proportion of women with unmet needs, the persistence of wealth-related disparities continues to be a challenge, and changes are needed to help women who face substantial barriers to accessing contraception. Although mechanisms are in place, better coordination between national and local government, as well as civil society organizations, remains challenging. In particular, the Philippines’ national government is unable to rally strategic support from government agencies outside the health sector or from civil society organizations.^30^ As a response, the national government could consider including family planning as part of a holistic approach towards the population’s well-being.^31^

Generally, access to contraceptives in lower-middle-income countries like the Philippines is constrained by multiple factors, from a lack of information and the fear of potential contraceptive side-effects to inadequate financial resources and limited reproductive health services.^32^^,^^33^ Women with a lower socioeconomic status stand to benefit most from initiatives that address barriers to accessing contraception. The public sector could narrow gaps in access between different socioeconomic groups by ensuring that health facilities are in easily accessible locations and that outreach services are provided by health workers.

An inability to rally support from local government can lead to missed opportunities. As local government provides the primary point of contact between the population and the health-care system, it plays an important role in reducing unmet needs contraception needs. By increasing their supply and by public health campaigns that correct misperceptions about contraceptives, local governments can foster a more supportive environment for modern family planning methods. In addition, community health workers could play a more active role, given that they enjoy the trust of community members.^34^ Effective policies should also include a call for strategies that address the need to diversify the types of contraceptive methods available and to ensure their availability, especially as many facilities, both globally and in the Philippines, often run out of stocks of various contraceptives.^29^^,^^35^ An effective mechanism for the timely updating and restocking of supplies could help avoid shortages. Complementary initiatives, such as counselling, that are sorely lacking in many jurisdictions could also promote the effective application of family planning methods.^36^

Sustainable financing plays a central role in addressing wealth-based disparities in the unmet need for contraception. Given that globally, an estimated 77% of women who wish to avoid pregnancy use modern methods of contraception (a figure that has risen substantially over the years), removing cost barriers through government financing of contraceptives is integral to ensuring access and to closing the wealth-based gap.^37^ Moreover, the recent decline in donor funding for family planning necessitates a stronger commitment from governments and domestic stakeholders to ensure that the increasing demand for contraceptives is met, and that the almost US$ 70 billion estimated to be required to tackle the unmet need for family planning in 120 countries between 2020 and 2030 is made available.^38^^,^^39^

Several limitations must be noted in interpreting our findings. First, the unmet need for contraception is self-reported and based on recall. Second, in the absence of a variable associated with the policy, our analysis of how the change in unmet needs was related to the Responsible Parenthood and Reproductive Health Law was performed on an intention-to-treat basis. Given that the law was fully implemented after 2017, as indicated by official documents and by observable changes in the Philippine government’s actions, we posit that changes after 2017 can be partially explained by health policy. Finally, as policy-making was centralized and policy was implemented nationally, no control group was available, which limited our study’s ability to establish a causal relationship.

Moving forward, future studies could examine trends in unmet needs for specific types of contraceptive and reasons for hesitancy in adopting certain methods. There is also a need to explore gaps in policy implementation and, particularly, the effectiveness of local government.

The broader implications of the Responsible Parenthood and Reproductive Health Law for socioeconomic development remain to be seen. Of particular concern is the greater proportion of women of a low socioeconomic status who have an unmet need for contraception as this could lead to more unplanned pregnancies. After all, the more reproductive health policy can reduce barriers to accessing contraceptives, facilitate their effective use and help prevent unintended pregnancies, the greater the impact on poverty reduction and human development.
